# Jejuno-jejunal invagination caused by epithelioid sarcoma: a case report

**DOI:** 10.1186/1752-1947-3-89

**Published:** 2009-10-30

**Authors:** Ana Bento, Hamilton Baptista, Carlos Pinheiro, Fernando Martinho

**Affiliations:** 1 Department of Surgery, Surgery II, University Hospital of Coimbra, Praceta Mota Pinto, 3000 Coimbra, Portugal

## Abstract

**Introduction:**

Jejuno-jejunal invagination is a rare condition and is usually caused by a benign lesion. We describe the case of a patient with a jejunal epithelioid sarcoma. Epithelioid sarcoma is a rare histologic subtype of sarcoma and few cases have been published.

**Case presentation:**

A 70-year-old Caucasian man presented with vomiting and anemia. A jejuno-jejunal invagination was diagnosed and the patient underwent surgery. An epithelioid sarcoma of the wall of the jejunum was found on the invaginated ansa.

**Conclusion:**

To the best of our knowledge, an epithelioid sarcoma has never been reported to arise at the wall of the proximal jejunum or to present with jejuno-jejunal invagination.

## Introduction

Adult jejuno-jejunal invagination is unusual [1] and it presents as a high bowel occlusion and/or obstruction requiring urgent surgical treatment. Usually the cause is a benign lesion of the jejunal wall. Neoplastic disease seldom occurs in the small bowel and even amongst those cases, sarcoma is infrequent. To the best of our knowledge, no published case has described an invagination caused by sarcoma.

Epithelioid sarcoma (ES) is a distinct clinicopathologic entity. This tumor is a relatively rare soft tissue neoplasm of unknown histogenesis. It is usually a slow-growing tumor and mostly occurs in the distal extremities of young adults. Both local recurrence and metastasis are common [2]. An aggressive subtype of ES has been identified known as 'proximal-type/axial-type' [2].

## Case presentation

A 70-year-old Caucasian man, who was being treated for a *Staphylococcus aureus *infection of a hip prosthesis in the Orthopaedic Surgery Department, was found to have anemia. His skin was pale, blood pressure was 90/60 mmHg, hemoglobin (Hb) had dropped to 9.4 g/dl (normal: 13.017.0 g/dl) but his mean corpuscular volume was normal.

A few days later, the patient complained of nausea and vomiting after meals; his Hb had decreased to 7.6 g/dl and he was given a blood transfusion. A fecal occult blood test was positive, an abdominal X-ray was unremarkable showing no air fluid levels, and an endoscopic examination of his stomach was inconclusive, showing only food remains. Bilious postprandial vomiting ensued and melena appeared. The patient's Hb level continued to fall, and repeated blood transfusions were required. A new endoscopic examination of his stomach was scheduled to evaluate his duodenum, but no pathology was found. Total colonoscopy, including visualization of the terminal ileum, was performed but found no lesions. The patient developed severe vomiting.

**Figure 1:**
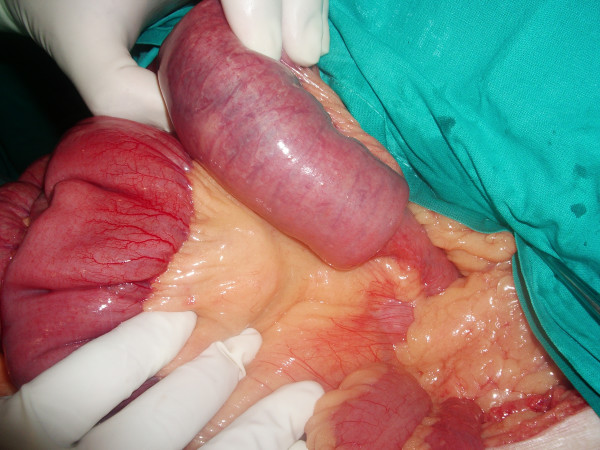
**Invaginated proximal jejunal ansa (10 cm long)**.

An abdominal computed tomography (CT) scan was carried out, revealing one thickened ansa in the proximal jejunum, which suggested an invagination. There was also intense intravenous contrast uptake at this location (Figure 1).

**Figure 2:**
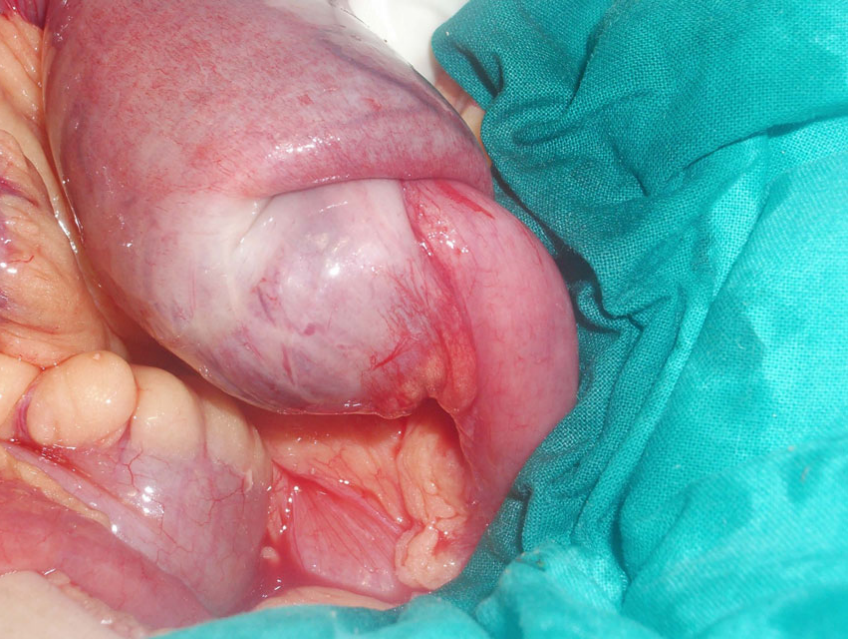
**The proximal jejuno-jejunal invagination is shown to be quite close to the angle of Treitz**.

**Figure 3:**
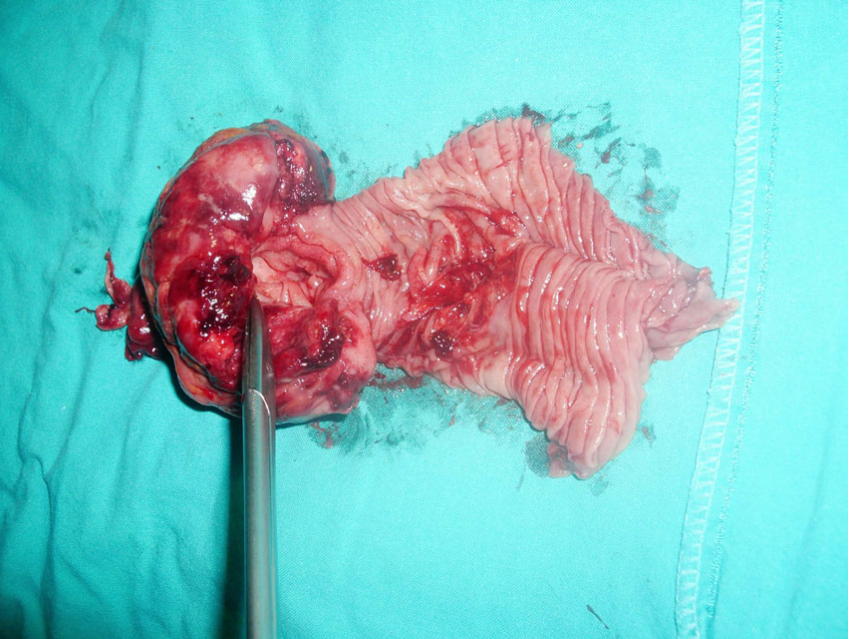
**The removed section was opened and shown to contain a tumor**.

The patient underwent laparotomy and, during surgery, it was discovered that in fact a jejuno-jejunal invagination was present at the location of an intestinal tumor. Reduction was impossible and resection was performed, followed by an end-to-lateral mechanic anastomosis with CEEA 25 (Figure 2 and Figure 3).

**Figure 4:**
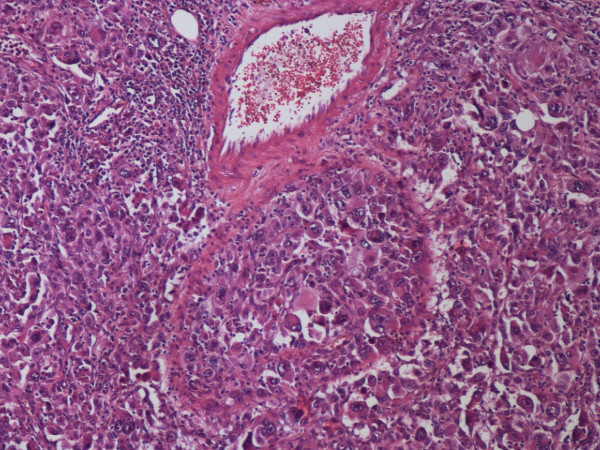
**Large cells with bizarre nuclei and abundant granulocytic infiltrate (hematoxylin and eosin, 200×)**.

**Figure 5:**
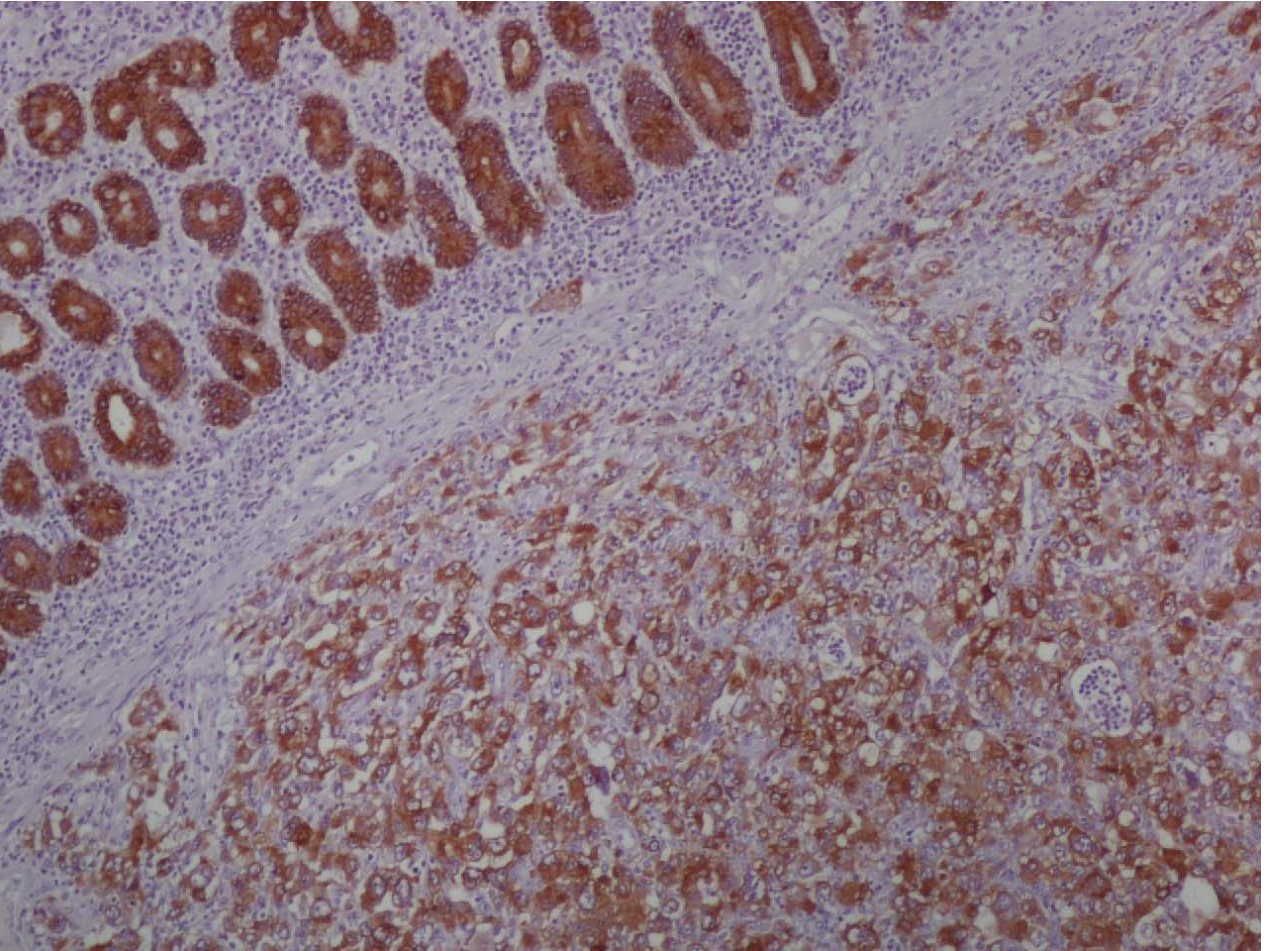
**AE 1/AE 2**.

In the anatomopathological examination, a malignant neoplasm was found. It had undifferentiated characteristics, large cells with bizarre nuclei and abundant granulocytic infiltrate. Histochemical markers revealed epithelioid features (Table 1) of an undifferentiated tumor, pointing to ES (Figure 4 and Figure 5).

**Table 1:** Immunohistochemical profile of the tumor

Desmin	Absent
S 100	Absent
CD 31	Absent
CD 34	Absent
CD 117	Absent
CD 68	Absent
F VII	Absent
Vimentin	Present
MNF 116	Present
AE 1/AE 3	Present
CAM 5.2	Present

Serum tumor marker levels for carcinoembryonic antigen (CEA) were normal, but CA 125 was elevated (41 U/ml; normal <27 U/ml). Both the thoraco-abdominopelvic CT scan performed with intravenous contrast and the positron emission tomography (PET), performed 1 month after surgery revealed no remaining or new lesions.

## Discussion

Less than 3% of all gastrointestinal invaginations occur in adult patients and the most common location on the small bowel is at its distal end, either ileo-ileal or ileo-colic. Principal symptoms are diffuse abdominal pain, nausea, vomiting and interruption of stool and gas emissions. Melena and hematochezia may be present.

Clinical presentation may be acute, with complete mechanical obstruction, eventually with strangulating injury, or chronic, with relapsing abdominal pain, relieved by spontaneous reduction. In some instances, the condition has been present for months or years at the time of diagnosis. Frequently, the diagnosis is made intra-operatively, on the occasion of urgent exploratory laparotomy performed for obstruction, hemorrhage or perforation.

The abdominal CT scan has both the highest sensitivity and specificity in this situation. Longitudinal images are likened to a 'sandwich' and transverse ones to 'bulls' eyes' or 'doughnuts'.

It has been estimated that 80 to 90% of all intestinal invaginations occur at the location of a structural lesion of the bowel wall and 14% of those are malignant tumors. Even though it is the longest segment of the digestive system, small bowel tumors represent no more than 3% of all gastrointestinal neoplasms [3,4]. Malignant small bowel tumors are adenocarcinomas in 35% of cases, carcinoid tumors in 28%, lymphoma in 21% and sarcoma in 10% [3,5]. Only 2% of adult sarcomas are located in the gastrointestinal system. The literature on such a rare pathology is notoriously scarce.

Undifferentiated tumors are those that lack microscopic features of tissue differentiation. Further study of these lesions has been enhanced in recent years by increasingly available immunohistochemical assays, which often allow some differentiation to be established [6].

Since there is no specific marker, the rather challenging diagnosis of ES was reached in our patient by combining the evidence and ruling out more frequent candidates. Epithelioid differentiation was established by the presence of low molecular weight cytokeratins, namely CK 8, CK 18 and CK 19 which cause CAM 5.2 antibody positivity, and by a mixture of several keratins signaled by the AE 1/AE 3 antibody [6].

ES is a rare soft tissue sarcoma with a known tendency for local recurrence, originally described by Enzinger in 1970 [7-9]. Two principal variants are known to occur: the conventional distal type and the more recently described proximal type. The distal type, which was the first to be described, is mostly seen in the distal extremities (commonly the hands) of young adults. On histomorphology, it displays a typical 'granuloma-like' appearance [7]. Fibrous, histiocytoma-like and angiomatoid subtypes have also been noted.

Lately, an aggressive subtype of ES has increasingly been recognized, the 'proximal-type/axial-type' [7,8] described in 1997 by Guillou. It mostly affects older individuals, often in axial or deep locations, displays a prominent atypical and pleomorphic appearance and an aggressive clinical behavior with poor outcome [9]. However, very few perivisceral ES have been described and they are mainly related to the colon and bladder in pelvic and perineal sites [9,10]. The histogenetic origin of ES is unknown. Immunohistochemical studies have been performed by previous investigators to characterize this tumor.

Local surgical resection is the single consistently proposed treatment and it need not include lymphadenectomy, since this has not been shown to influence prognosis [11]. The documented tendency for local relapse and pulmonary metastasis warrants prolonged clinical and radiological postoperative screening.

## Conclusion

This is an extremely unusual clinical case where a rare tumor, an epithelioid sarcoma ('proximal-type/axial-type'), was found at the proximal jejunum, a location where it had not previously been described. Furthermore, it presented with jejuno-jejunal invagination, which is also uncommon.

## Abbreviations

CT: computed tomography; CEA: carcinoembryonic antigen; ES: epithelioid sarcoma; Hb: hemoglobin; JI: jejunum invagination; PET: positron emission tomography; UT: undifferentiated tumor.

## Consent

Written informed consent was obtained from the patient for publication of this case report and any accompanying images. A copy of the written consent is available for review by the Editor-in-Chief of this journal.

## Competing interests

The authors declare that they have no competing interests.

## Authors' contributions

AB performed the pre-operative study, took part in the surgery, followed the patient, reviewed the literature and was a major contributor in writing the manuscript. HB performed the surgery and reviewed the manuscript. CP participated in the literature review. FM reviewed the final text. All authors read and approved the final manuscript.
